# Supplementary material for: SARS-CoV-2 infection dynamics in a MHCI-mismatched lung transplant recipient
Source: Nat Commun. 2025 Sep 16;16:8292. doi: 10.1038/s41467-025-63681-y (PMC12441153; doi:10.1038/s41467-025-63681-y)
Supplement: Supplementary file 1 — Supplementary Information [file 41467_2025_63681_MOESM1_ESM.pdf]

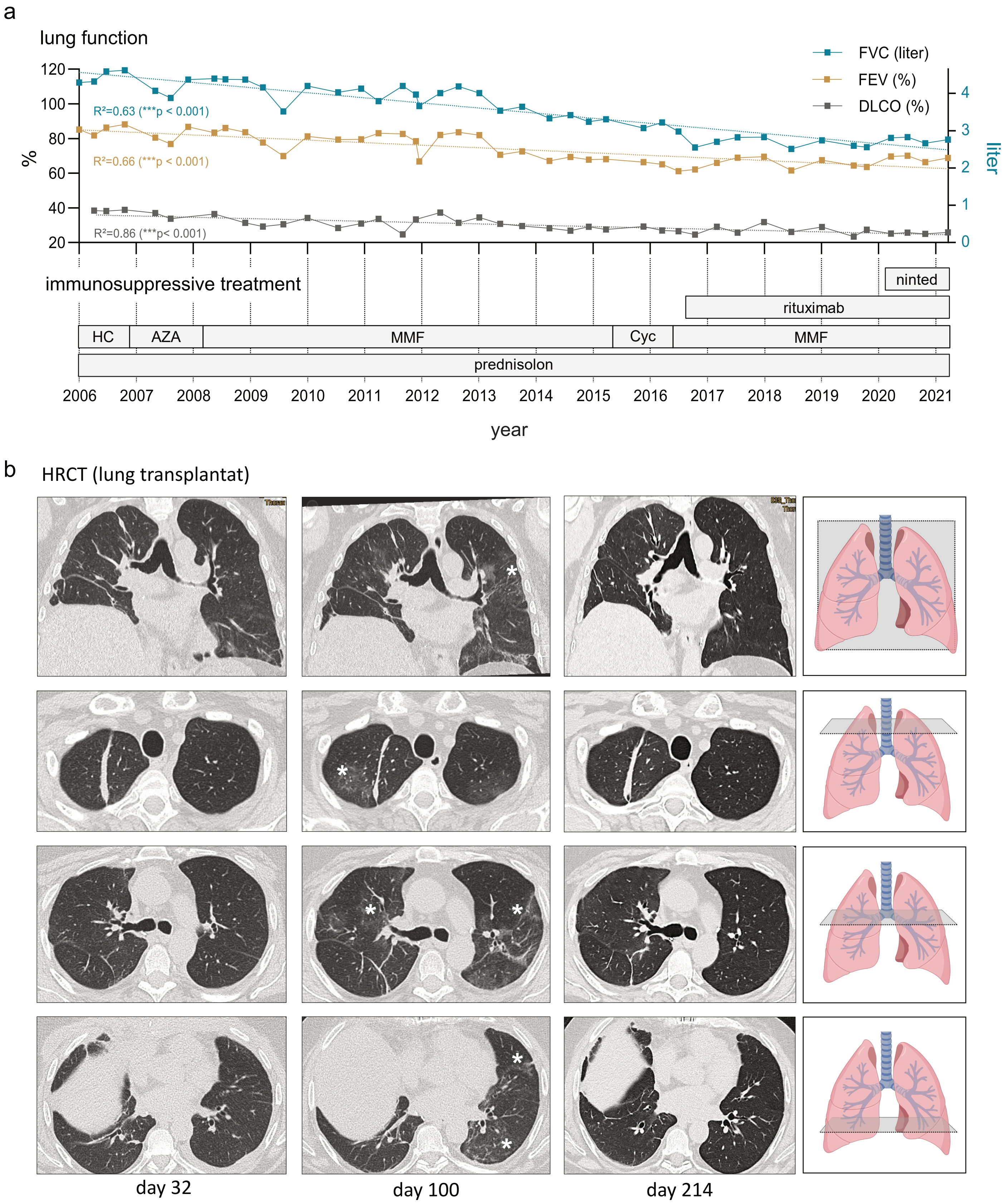

Supplementary Figure 1. Lung function, immunosuppressive drugs prior to lung transplantation and high-resolution computed chest tomography (HRCT) post transplantation. (a) Temporal overview of the lung function and immunosuppressive regimen prior lung transplantation. **(b)** HRCT of different sectional planes (depicted on the right) of the transplant 32, 100 and 214 days after lung transplantation. At day 100, ground glass opacities (asteriks) indicate inflammation attributed to SARS-CoV-2 infection, whereas similar alterations were not present at day 32 and 214. The right column schematically depicts the lungs with trachea in blue, lungs in red and airway in light blue. Slice planes are also indicated. Lung icon adapted from BioRender. Fuchs (2025) https://BioRender.com/hv8kbn1.

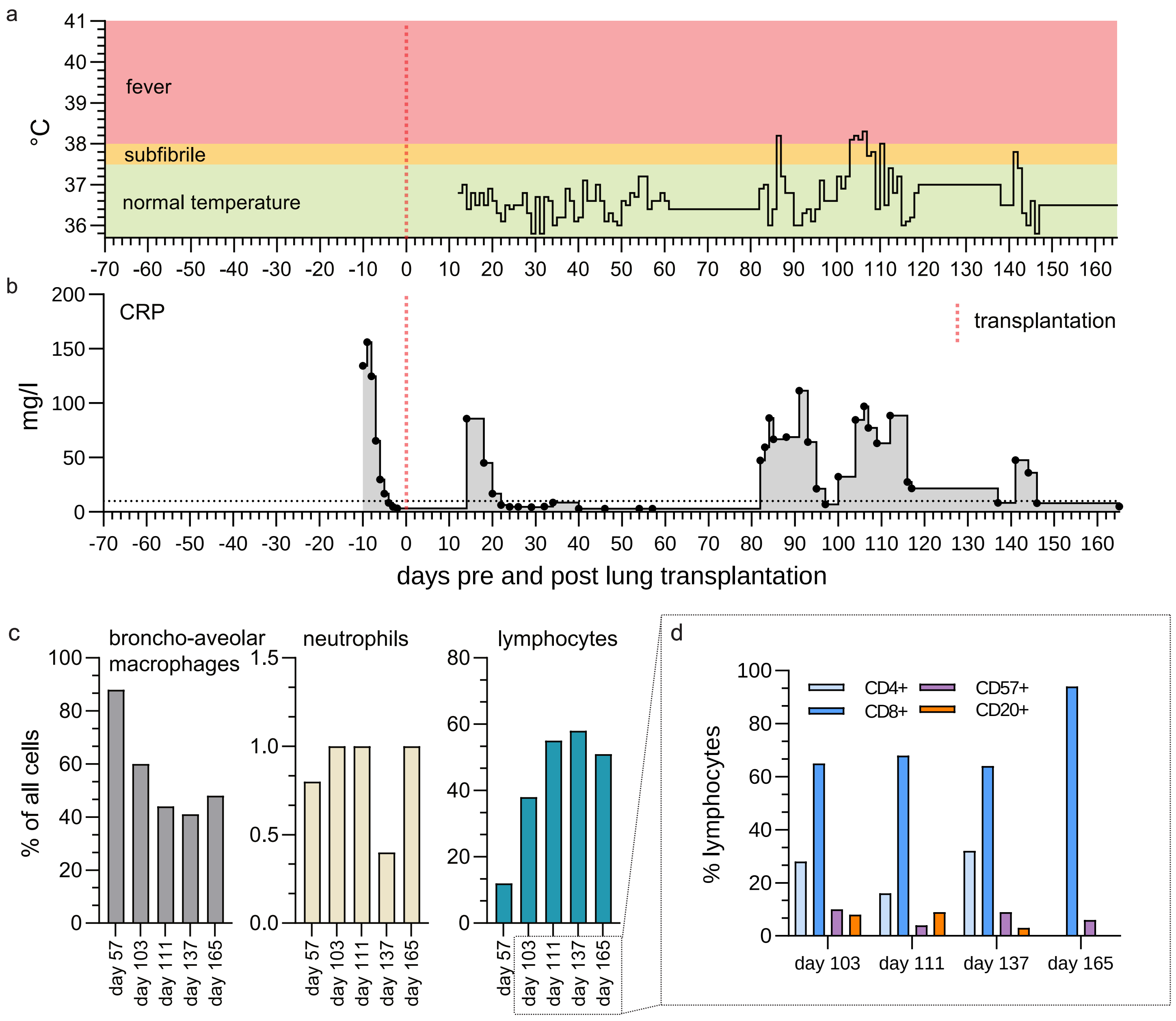

Supplementary Figure 2. Clinical parameters and immune cell counts. (a, b) Temporal overview of (a) temperature and (b) CRP blood levels. **(c)** Immune cell counts of BAL at different time points post lung transplantation and **(d)** differential analysis of CD4+, CD8+, CD57+ and CD20+ lymphocytes in BAL.

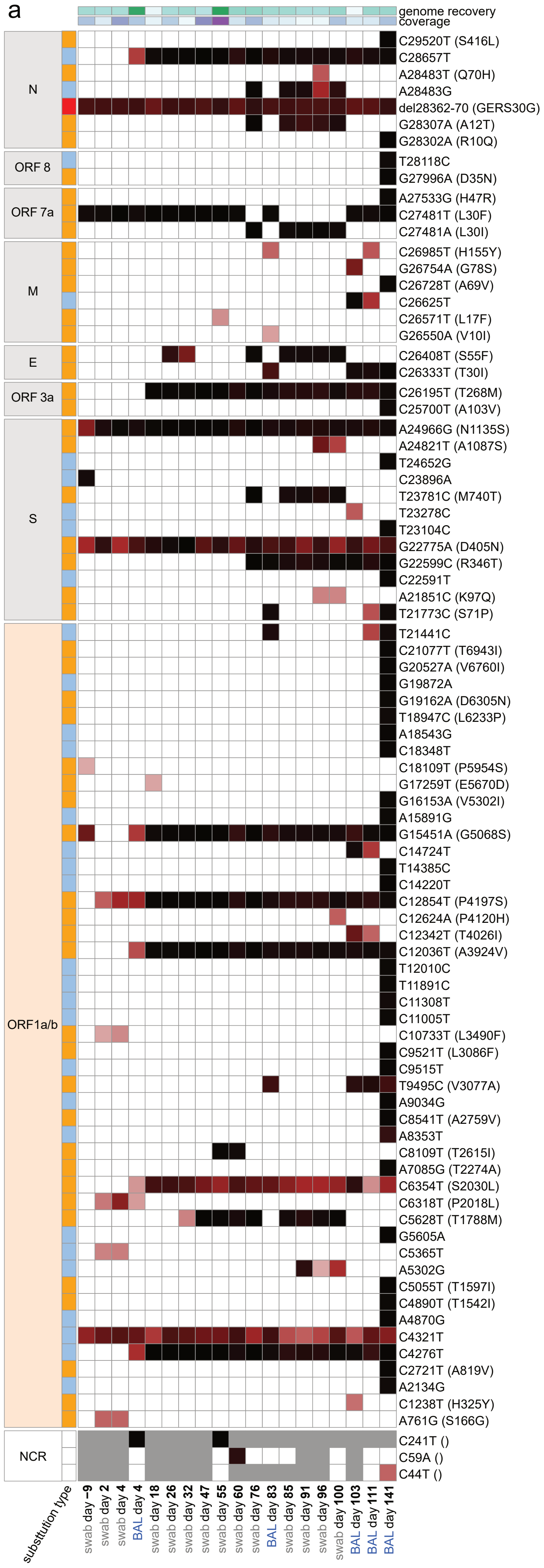

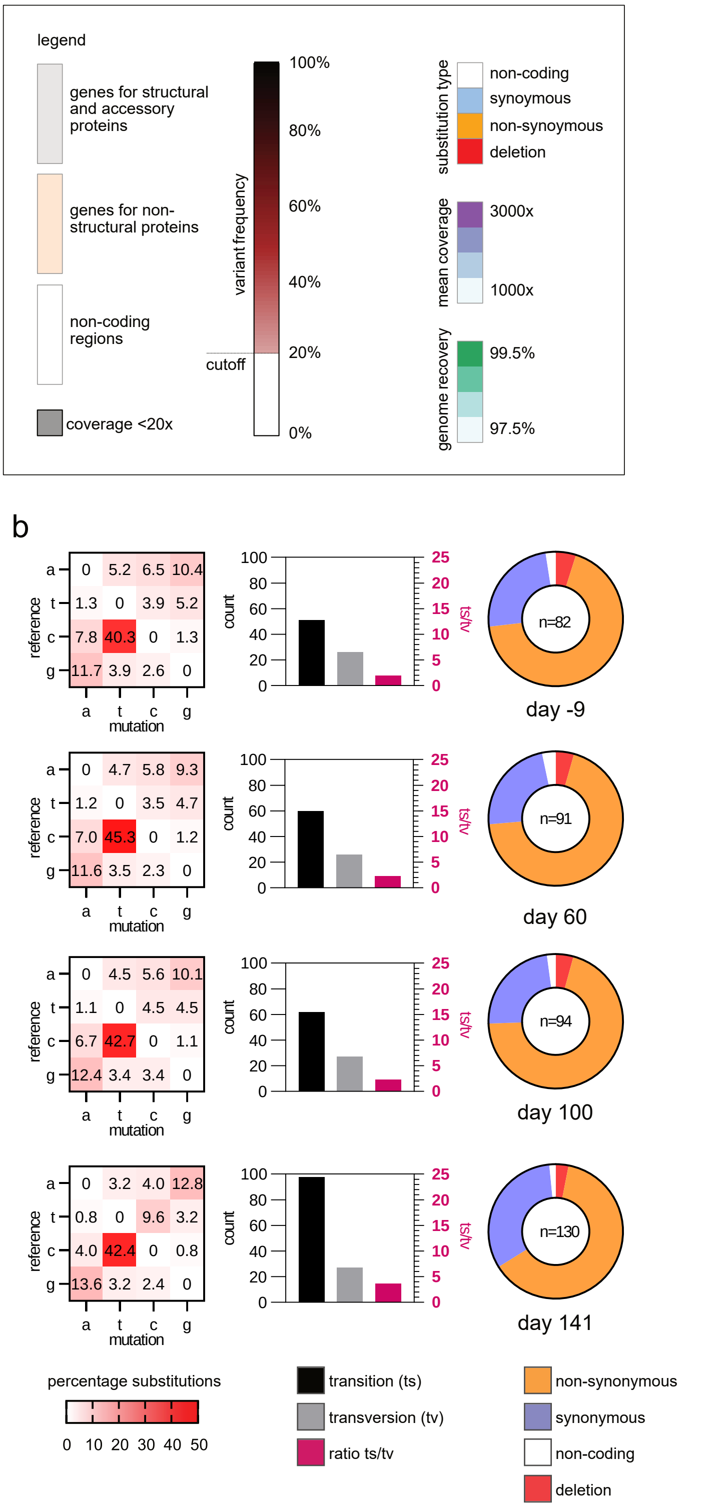

Supplementary Figure 3. Variant frequency and mutational analysis of full genome SARS-CoV-2 Illumina sequencing results during the time of infection. Mutations were detected in comparison to the Wuhan-Hu-1 reference sequence. **(a)** The heatmap summarizes the positions in the viral genome and the variant frequencies in the different samples (shown are values above 20%) that are not common to all samples (defined as mutations with a higher variant frequency of 70% in all samples). The days of sampling are indicated at the bottom (nasopharyngeal swabs (grey), BAL (blue)), heatmap color intensity indicates variant frequencies, left are mutational effects and gene products indicated and on the top overall genome recovery and mean coverage. **(b)** Percentage of substitutions (left), substitution type (middle) and amino acid effect (right) of novel viral mutations with a variant frequency > 50% compared to Wuhan-Hu-1.

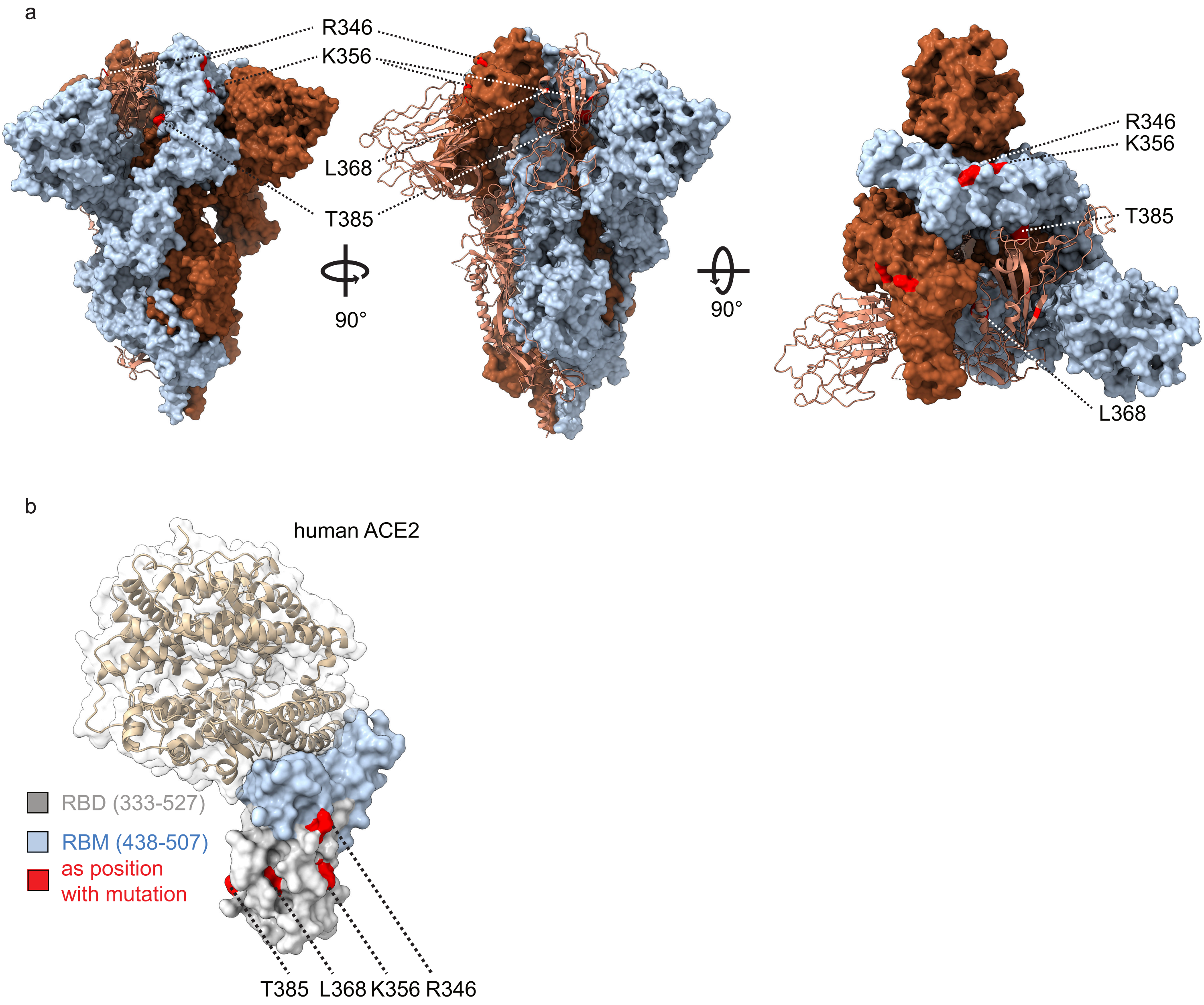

Supplementary Figure 4. Structural analysis of the viral spike. 3D representations of **(a)** the full-length viral Omicron BA.2 variant spike (pdb accession: 7XIX) and **(b)** the receptor-binding domain (RBD) of Omicron BA.2 variant in complex with the human ACE2 (pdb accession: 7XB0). The mutational sites at position R346, K356, L368 and T385 are highlighted in red.

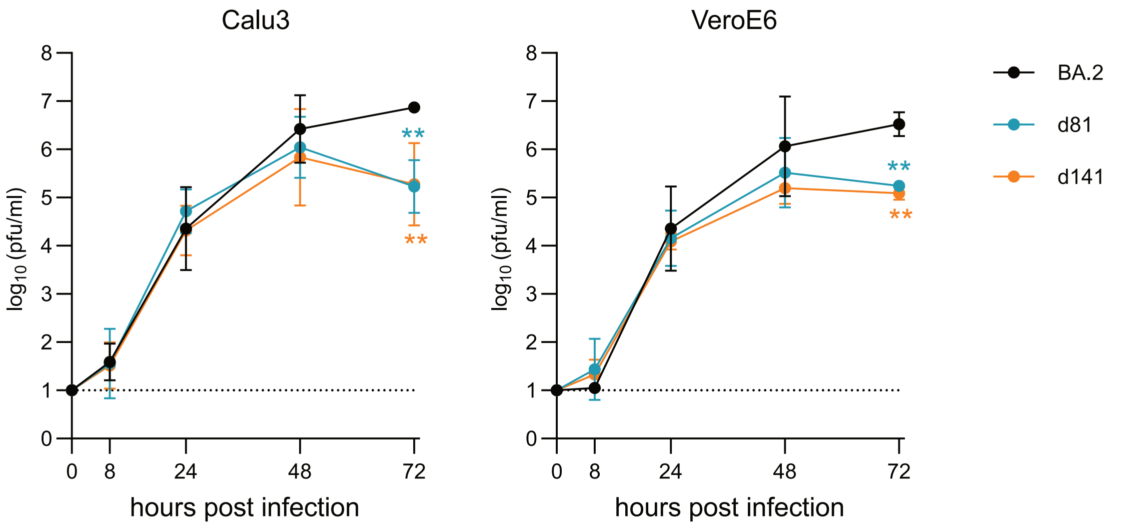

Supplementary Figure 5. Viral growth kinetics in Calu3 and VeroE6 cells of the patient isolates compared to a prototypic BA.2 isolate. Cells were infected at a multiplicity of infection (MOI) of 0.001. At 8, 24, 48 and 72 h post infection, cell culture supernatants were collected and viral titers were determined by plaque assay. The log-transformed titers are shown as means ± SD of results from three independent experiments. Dotted lines indicate the assay cut-off. Significance was determined via two-way ANOVA with a Sidak´s multiple comparison test (**p < 0.01).

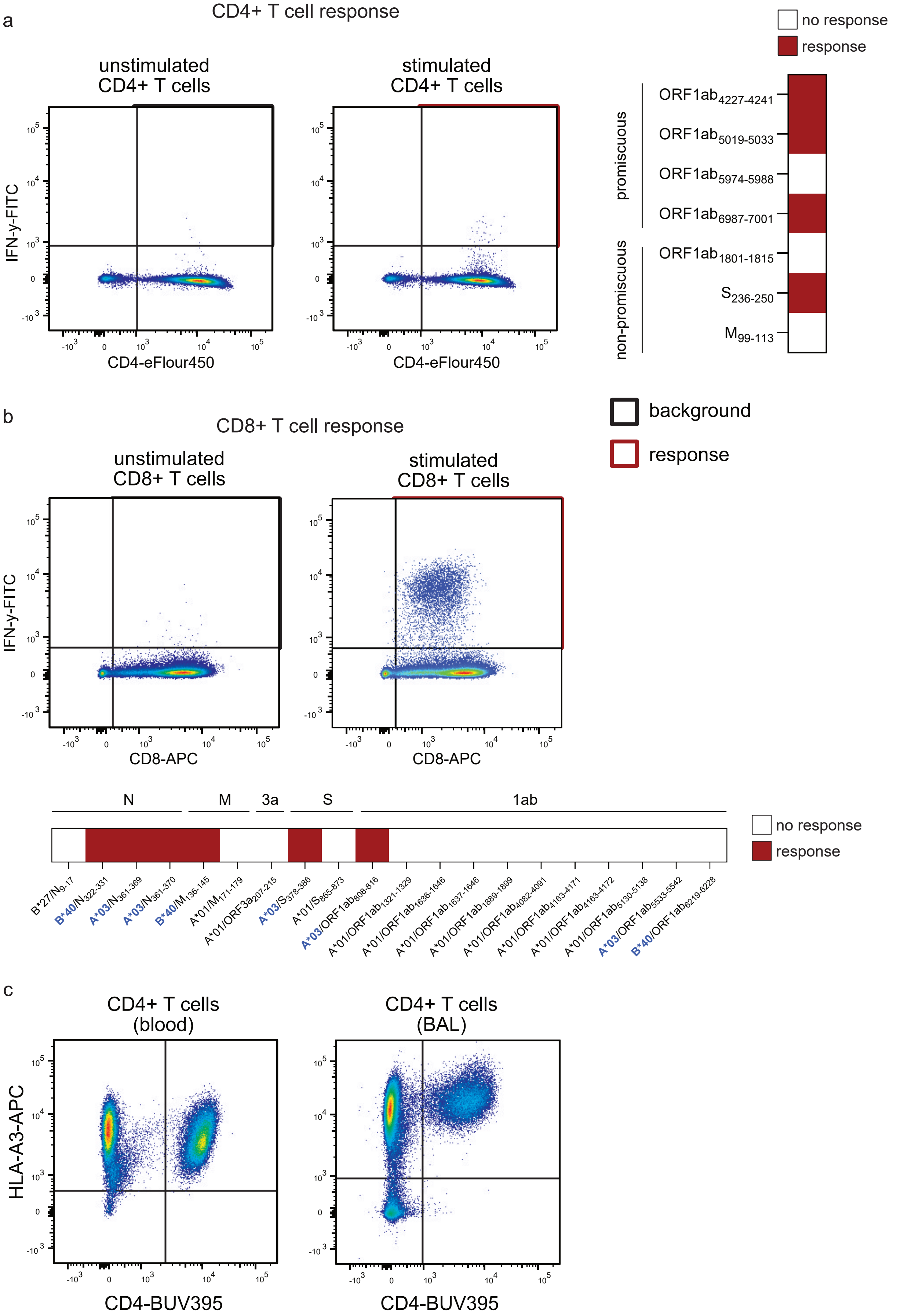

Supplementary Figure 6. SARS-CoV-2-specific CD4+ and CD8+ T cell responses in blood samples of lung transplant recipient. (a) Exemplary dot plots depicting IFN-γ-release of unstimulated and peptide-stimulated CD4+ T cells after *in vitro* expansion. Circulating CD4+ T cell responses are shown against epitopes that are restricted by the HLA-DRB1 types (non-promiscuous) of the lung transplant recipient as well as against promiscuous peptides. T cell response was determined by the difference between the signal of stimulated (red square) and the signal of unstimulated (black square) samples with a subsequent cut-off of 0.01%. **(b)** Exemplary dot plots depicting IFN-γ-release of unstimulated and peptide-stimulated CD8+ T cells after *in vitro* expansion. CD8+ T cell responses are presented against immunodominant epitopes described to be restricted by the HLA-A/B types of the lung transplant recipient (A*03, B*40) and the lung donor (A*01, B*27). Recipient HLA-A/B are indicated in blue. T cell response was determined by the difference between the signal of stimulated (red square) and the signal of unstimulated (black square) samples with a subsequent cut-off of 0.01%. **(c)** Exemplary dot plots showing HLA-A3+ (recipients') CD4+ T cells in blood and BAL of lung transplant recipient.

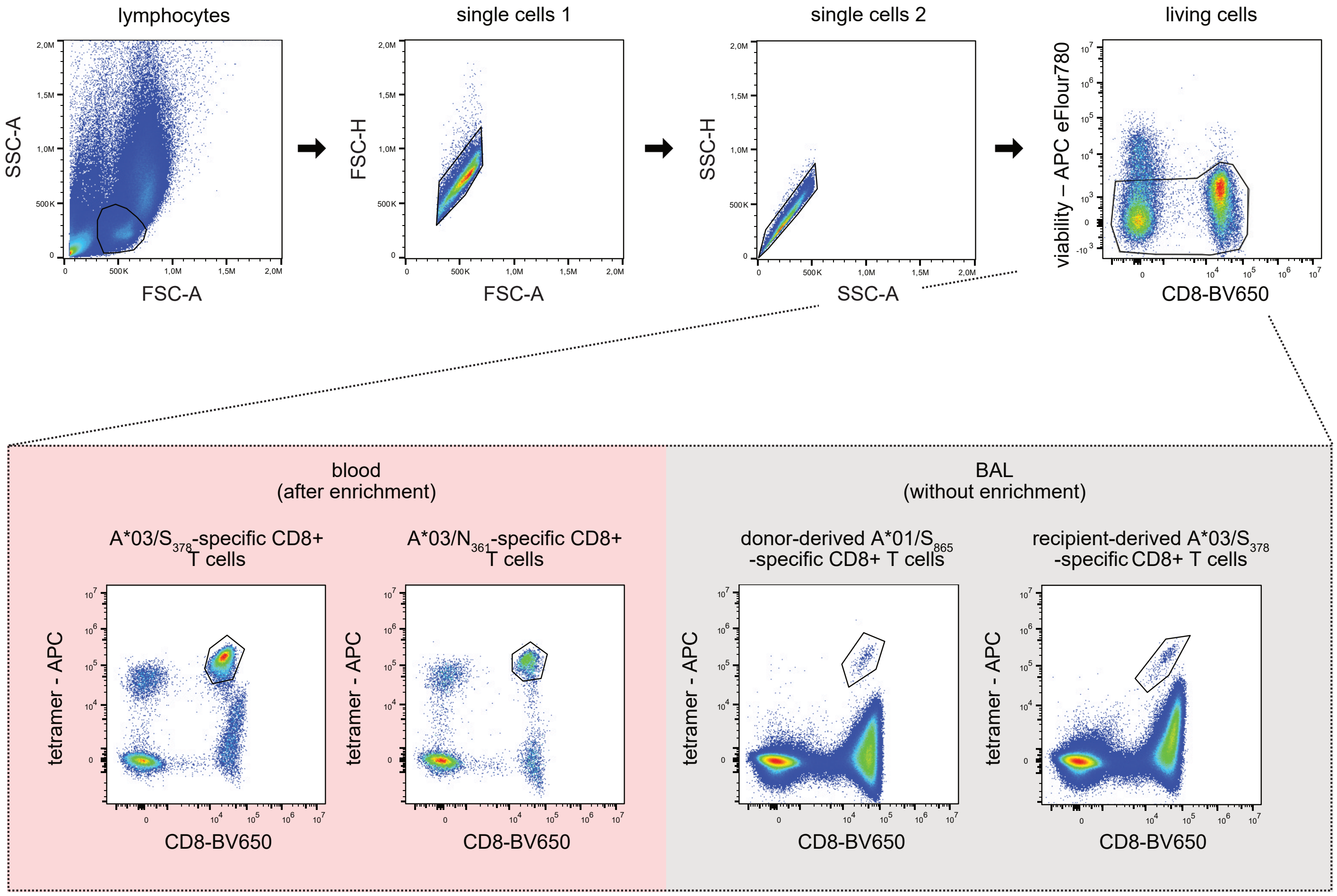

Supplementary Figure 7. Gating strategy. Gating strategy of flow cytometry data. SARS-CoV-2-specific CD8+ T cells in the blood were detected via pMHCI tetramer-based enrichment and in BAL samples via *ex vivo* pMHCI tetramer staining. Dot plots show recipient-derived A*03/S_{378}- and A*03/N_{361}-specific CD8+ T cells in the blood as well as lung donor (A*01/S_{865})- and recipient (A*03/S_{378})-derived CD8+ T cells in BAL samples of the lung transplant recipient 1 day before viral clearance.

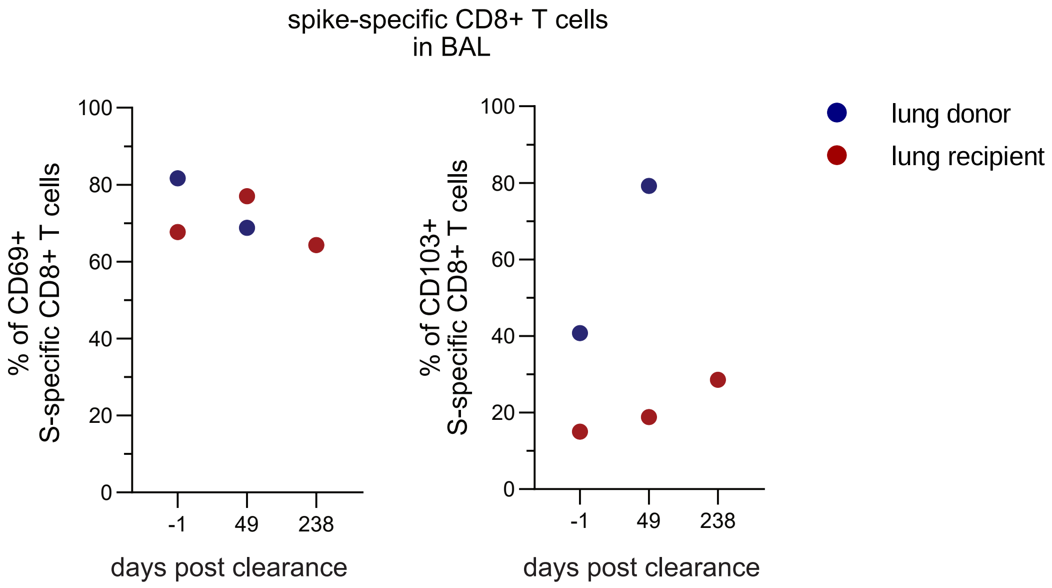

Supplementary Figure 8. Expression of tissue-resident markers on spike-specific CD8+ T cells in BAL samples. CD69 and CD103 expression of recipient (A*03/S_{378} (red)) - and donor (A*01/S_{865} (blue))-derived, spike-specific CD8+ T cells detected in BAL samples 1 day before and 49 and 238 days after complete viral clearance.

a

CD8+ T cell epitopes

	B*27/N_{9}	B*40/N_{322}	A*03/N_{361}	B*40/M_{136}	A*01/M_{171}	A*03/S_{378}	A*01/S_{865}
	QRNAPRITF	MEVTPSGTWL	KTFPPTEPKK	SELVIGAVIL	ATSRTLSYY	KCYGVSPTK	LTDEMIAQY
Day 2	....L....	....	....	....	....	.......I.	....
Day 18	....L....	....	....	....	....	.......I.	....
Day 26	....L....	....	....	....	....	.......I.	....
Day 32	....L....	....	....	....	....	.......I.	....
Day 47	....L....	....	....	....	....	.......I.	....
Day 55	....L....	....	....	....	....	.......I.	....
Day 60	....L....	....	....	....	....	.......I.	....
Day 76	....L....	....	....	....	....	.......I.	....
Day 83	....L....	....	....	....	....	.......I.	....
Day 85	....L....	....	....	....	....	.......I.	....
Day 91	....L....	....	....	....	....	.......I.	....
Day 96	....L....	....	....	....	....	.......I.	....
Day 103	....L....	....	....	....	....	.......I.	....
Day 111	....L....	....	....	....	....	.......I.	....

	A*01/$ORF3a_{507}$	A*03/$ORF1ab_{808}$	A*01/$ORF1ab_{1321}$	A*01/$ORF1ab_{1636}$	A*01/$ORF1ab_{1889}$	A*03/$ORF1ab_{5533}$	B*40/$ORF1ab_{6219}$
	FTSDYYQLY	VTNNTFTLK	PTDNYITTY	HTTDPSFLGRY	CTEIDPKLDNY	VVYRGTTTYK	IEYPIIGDEL
Day 2	....	....	....	....	....	....	....
Day 18	....	....	....	....	....	....	....
Day 26	....	....	....	....	....	....	....
Day 32	....	....	....	....	....	....	....
Day 47	....	....	....	....	....	....	....
Day 55	....	....	....	....	....	....	....
Day 60	....	....	....	....	....	....	....
Day 76	....	....	....	....	....	....	....
Day 83	....	....	....	....	....	....	....
Day 85	....	....	....	....	....	....	....
Day 91	....	....	....	....	....	....	....
Day 96	....	....	....	....	....	....	....
Day 103	....	....	....	....	....	....	....
Day 111	....	....	....	....	....	....	....

	A*01/$ORF1ab_{4082}$	A*01/$ORF1ab_{4163}$	A*01/$ORF1ab_{5130}$
	NTCDGTTFTY	CTDDNALAYY	DTDFVNEFY
Day 2	....	....	....
Day 18	....	....	....
Day 26	....	....	....
Day 32	....	....	....
Day 47	....	....	....
Day 55	....	....	....
Day 60	....	....	....
Day 76	....	....	....
Day 83	....	....	....
Day 85	....	....	....
Day 91	....	....	....
Day 96	....	....	....
Day 103	....	....	....
Day 111	....	....	....

b

CD4+ T cell epitopes

	DRB1*04/$ORF1ab_{1801-1815}$	$ORF1ab_{4227-4241}$	$ORF1ab_{5019-5033}$	$ORF1ab_{5974-5988}$	$ORF1ab_{6987-7001}$	DRB1*03/$S_{236-250}$	DRB1*04/M_{99-113}
	ESPFVMMSAPPAQYE	YLYFIKGLNNLNRGM	PNMLRIMASLVLARK	MTYRRLISMMGFKMN	WWTAFVTNVNASSSE	TRFQTLLALHRSYLT	SFRLFARTRSMWSFN
Day 2	....	....	....	....	....	....	....
Day 18	....	....	....	....	....	....	....
Day 26	....	....	....	....	....	....	....
Day 32	....	....	....	....	....	....	....
Day 47	....	....	....	....	....	....	....
Day 55	....	....	....	....	....	....	....
Day 60	....	....	....	....	....	....	....
Day 76	....	....	....	....	....	....	....
Day 83	....	....	....	....	....	....	....
Day 85	....	....	....	....	....	....	....
Day 91	....	....	....	....	....	....	....
Day 96	....	....	....	....	....	....	....
Day 103	....	....	....	....	....	....	....
Day 111	....	....	....	....	....	....	....

Supplementary Figure 9. Epitope sequences of the BAL-derived SARS-CoV-2 isolates. (a, b) Comparison of tested (a) CD8+ T cell and (b) CD4+ T cell epitope sequences with amino acid sequences of SARS-CoV-2 isolated from the patient at indicated time points.

Supplementary Table 1 Relevant drugs given to the patient (including the indication for its administration, its mode of action described in literature and the respective PMIDs)

Drug	Indication in this manuscript	Mode of action	PMIDs
Rituximab	Scleroderma-associated interstitial lung disease	B-cell-depleting antibody	37772987, 14576843
Mycophenolat Mofetil	Scleroderma-associated interstitial lung disease<br><br>Immunosuppression after lung transplantation	Reversible, non-competitive inhibitor of inosine-5′-monophosphate (especially B- and T-cell reducing effects)	16251851, 4416724
Tocilizumab	Scleroderma-associated lung disease<br>SARS-CoV-2 infection	Interleukin-6-neutralizing antibody	35250575, 37773003
Prednisolon	Scleroderma-associated interstitial lung disease<br><br>Immunosuppression after lung transplantation	Upregulation of anti-inflammatory genes<br><br>Downregulation of pro-inflammatory genes<br>inhibition of prostaglandin synthesis<br><br>Inhibition of immune cell migration	20398732
Basiliximab	Immunosuppression (induction therapy) for lung transplantation	Interleukin 2-receptor antagonist, thereby blocking activity of IL-2 especially on T-cells.	39642952, 12437498, 10188761
Tacrolimus	Immunosuppression after lung transplantation	Prevention of NFAT dephosphorylation by inhibiting calcineurin via FKBP-12 binding, thereby inhibiting T-cell activation and cytokine production	34992841, 32355853, 8588225
Everolimus	Immunosuppression after lung transplantation	Inhibition of mTOR and thereby inhibiting immune cell proliferation and release of pro-inflammatory and pro-proliferative cytokines	16805720, 12457444, 34992841, 32355853
Remdesivir	Treatment of SARS-CoV-2 infection	Inhibition of RNA-dependent RNA-polymerase in SARS-CoV-2	32284326
Molnupiravir	Treatment of SARS-CoV-2 infection	Molnupiravir acts as a nucleotide analogue (Molnupiravir Triphosphate) being incorporated in the RNA leading to nucleotide transitions	33989635, 34381216
Sotrovimab	Treatment of SARS-CoV-2 infection	Monoclonal antibody directed against different epitopes within the receptor binding domain of the SARS-CoV-2-spike protein thereby leading to:<br>- Virus neutralization<br>- Inhibition of virus-entry into cells<br>- Antibody-mediated phagocytosis and cytotoxicity	32422645, 36307535
Cilgavimab + Tixagevimab	Treatment of SARS-CoV-2 infection	Two monoclonal antibodies directed against different epitopes within the receptor binding domain of the SARS-CoV-2-spike protein thereby leading to:<br>- Virus neutralization<br>- Inhibition of virus-entry into cells<br>- Antibody-mediated phagocytosis and cytotoxicity	36307535

Supplementary Table 2 Common mutations present in all patient sequences in a frequency >70%

position	effect	gene	BA.2 defining
del510-18 (C900T)	deletion	ORF1ab	no
T670G (S135R)	non-syn	ORF1ab	yes
C900T (S212L)	non-syn	ORF1ab	no
G1980T (G572V)	non-syn	ORF1ab	no
C2790T (T842I)	non-syn	ORF1ab	yes
C3037T (F924)	syn	ORF1ab	yes
G4184A (G1307S)	non-syn	ORF1ab	yes
C9344T (L3027F)	non-syn	ORF1ab	yes
A9424G (V3053)	syn	ORF1ab	yes
C9534T (T3090I)	non-syn	ORF1ab	yes
C9866T (L3201F)	non-syn	ORF1ab	yes
C10029T (T3255I)	non-syn	ORF1ab	yes
C10198T	syn	ORF1ab	yes
G10447A	syn	ORF1ab	yes
C10449A (P3395H)	non-syn	ORF1ab	yes
del11288-96 (SGF3675)	deletion	ORF1ab	yes
C12880T	syn	ORF1ab	yes
C14408T (P4720L)	non-syn	ORF1ab	yes
C15714T	syn	ORF1ab	yes
C17410T (R5721C)	non-syn	ORF1ab	yes
A18163G (I5972V)	non-syn	ORF1ab	yes
C19955T (T6569I)	non-syn	ORF1ab	yes
A20055G	syn	ORF1ab	yes
C20719T	syn	ORF1ab	no
C21618T (T19I)	non-syn	S	yes
del21633-41 (LPPA24S)	deletion	S	yes
G21987A (G142D)	non-syn	S	yes
A22200G (V213G)	non-syn	S	yes
G22578A (G339D)	non-syn	S	yes
A22629C (K356T)	non-syn	S	no
C22664A (L368I)	non-syn	S	no
C22674T (S371F)	non-syn	S	yes
T22679C (S373P)	non-syn	S	yes
C22686T (S375F)	non-syn	S	yes
A22688G (T376A)	non-syn	S	yes
C22716T (T385I)	non-syn	S	no
A22786C (R408S)	non-syn	S	yes
C22792T	syn	S	no
G22813T (K417N)	non-syn	S	yes
T22882G (N440K)	non-syn	S	yes
G22992A (S477N)	non-syn	S	yes
C22995A (T478K)	non-syn	S	yes
A23013C (E484A)	non-syn	S	yes
A23040G (Q493R)	non-syn	S	yes
A23055G (Q498R)	non-syn	S	yes
A23063T (N501Y)	non-syn	S	yes
T23075C (Y505H)	non-syn	S	yes
A23403G (D614G)	non-syn	S	yes
C23525T (H655Y)	non-syn	S	yes
T23599G (N679K)	non-syn	S	yes
C23604A (P681H)	non-syn	S	yes
C23854A (N764K)	non-syn	S	yes
G23948T (D796Y)	non-syn	S	yes
A24424T (Q954H)	non-syn	S	yes
T24469A (N969K)	non-syn	S	yes
C25000T	syn	S	yes
C25584T	syn	ORF3a	yes
C26060T (T223I)	non-syn	ORF3a	yes
C26270T (T9I)	non-syn	E	yes
C26577G (Q19E)	non-syn	M	yes
G26709A (A63T)	non-syn	M	yes
C26801T	syn	M	no
C26858T	syn	M	yes
A27259C	syn	ORF6	yes
GAT27382CTC (D61L)	non-syn	ORF6	yes
C27549T	syn	ORF7a	no
C27807T	syn	ORF7b	yes
A28271T ()	non-coding	NCR	yes
C28311T (P13L)	non-syn	N	yes
GGG28881AAC (RG203KR)	non-syn	N	yes
A29510C (S413R)	non-syn	N	yes
del29734-29759	non-coding	NCR	no

Data Availability

GISAID Identifier: EPI_SET_230216es

doi: https://epicov.org/epi3/epi_set/230216es

Supplementary Table 3 List of antibodies. Each set was mixed in equal volumes. For staining, 5 µl of the mixture was used in two different stanings. Compensation was performed for every antibody in the respective set using compensation beads (B99883, Beckman Coulter) and stored as compensation matrix. These compensation matrix was then used to compensate the measurements.

Antigen	Conjugate	Clone	Dilution	Isotype	LOT number	Catalogue number	Manufacturer
CCR7	PE/Dazzle594	G043H7	1:50	Mouse IgG2a, κ	B348534	353236	BioLegend
CD8	BV650	RPA-T8	1:200	Mouse IgG1, k	B361475	301042	BioLegend
CD8	BV510	SK1	1:100	Mouse BALB/c IgG1, κ	4134058	563919	BD Biosciences
CD8	BV421	RPA-T8	1:200	Mouse IgG1, k	1292699	562428	BD Biosciences
CD8	APC	SK1	1:300	Mouse BALB/c IgG1, κ	4213691	345775	BD Biosciences
CD95	PE	DX2	1:16.67	Mouse IgG1, k	3114254	340480	BD Biosciences
CD95	BV421	DX2	1:16.67	Mouse IgG1, k	286661	562616	BD Biosciences
CD4	BUV395	SK3	1:100	Mouse IgG1, k	2332927	563550	BD Biosciences
CD4	eFlour450	RPA-T4	1:250	Mouse IgG1, k	2526326	48-0049-42	eBioscience
IFN-γ	FITC	25723.11	1:8	Mouse IgG2b	4260955	340449	BD Biosciences
TNF	PE-Cy7	MAb11	1:50	Mouse IgG1, κ	3292259	557647	BD Biosciences
CD107a	APC	H4A3	1:100	Mouse IgG1, k	5149854	560664	BD Biosciences
CD69	PE-Cy7	FN50	1:50	Mouse IgG1, k	2077748	25-0699-42	eBioscience
CD103	FITC	Ber-ACT8	1:20	Mouse IgG1	0079918-1	550259	BD Biosciences
HLA-A3	APC	GAP.A3	1:25	Mouse IgG2a	28968	17-5754	eBioscience
CD14	APC-eFluor780	61D3	1:400	Mouse IgG1, k	2730940	47-0149-42	eBioscience
CD19	APC-eFluor780	HIB19	1:400	Mouse IgG1, k	2892943	47-0199-42	eBioscience
viability dye	eFluor780		1:400		3154429	65-0865-14	eBioscience
viability dye	eFluor506		1:300		2220742	65-0866-14	eBioscience
**BAL 1**							
CD45	PE-Cy5	HI30	1:50	mouse IgG1 κ	811698	555484	BD Biosciences
CD3	FITC	UCHT1	1:50	mouse IgG1 κ	3222318	555332	BD Biosciences
CD4	APC	MEM-241	1:50	mouse IgG2b κ	85585	21270046X2	Immunotools
CD8	PE	UCHT-4	1:50	mouse IgG2a	620919	21620084X2	Immunotools
CD57	Pacific Blue	HNK1	1:50	mouse IgGM κ	B384454	359608	BioLegend
**BAL 2**							
CD45	PE-Cy5	HI30	1:50	mouse IgG1 κ	811698	555484	BD Biosciences
CD3	FITC	UCHT1	1:50	mouse IgG1 κ	3222318	555332	BD Biosciences
CD20	APC Fire 750	2H7	1:50	mouse IgG2b κ	B410442	302358	BioLegend
CD25	PE	M-A251	1:50	mouse IgG1 κ	9137985	555432	BD Biosciences
CD56	Pacific Blue	MEM188	1:50	mouse IgG2a κ	B405326	304629	BioLegend
HLA-DR	APC	LT-DR	1:50	mouse IgG2a	85585	21388996	Immunotools

Supplementary Table 4 GISAID accession numbers of BA.2 sequences from Freiburg Germany

seq	lineage	specimen
EPI_ISL_11030472	BA.2	swab
EPI_ISL_11030473	BA.2	swab
EPI_ISL_11030474	BA.2	swab
EPI_ISL_11268038	BA.2	swab
EPI_ISL_11268039	BA.2	swab
EPI_ISL_11268040	BA.2	swab
EPI_ISL_11268041	BA.2	swab
EPI_ISL_11268042	BA.2	swab
EPI_ISL_11793097	BA.2	swab
EPI_ISL_11268043	BA.2	swab
EPI_ISL_11793100	BA.2	swab
EPI_ISL_11793191	BA.2	swab
EPI_ISL_11793101	BA.2	swab
EPI_ISL_11793102	BA.2	swab
EPI_ISL_11793103	BA.2	swab
EPI_ISL_11793105	BA.2	swab
EPI_ISL_11793106	BA.2	swab
EPI_ISL_11793107	BA.2	swab
EPI_ISL_11793108	BA.2	swab
EPI_ISL_11793110	BA.2	swab
EPI_ISL_11793111	BA.2	swab
EPI_ISL_11793142	BA.2	swab
EPI_ISL_11793112	BA.2	swab
EPI_ISL_11793113	BA.2	swab
EPI_ISL_11793114	BA.2	swab
EPI_ISL_11793115	BA.2	swab
EPI_ISL_11793143	BA.2	swab
EPI_ISL_11793116	BA.2	swab
EPI_ISL_11793144	BA.2	swab
EPI_ISL_13748023	BA.2	swab
EPI_ISL_15409725	BA.2	swab
EPI_ISL_13748065	BA.2	swab
EPI_ISL_13748066	BA.2	swab
EPI_ISL_13748107	BA.2	swab
EPI_ISL_15739614	BA.2	swab
EPI_ISL_14935930	patient	swab day 2
EPI_ISL_15739616	BA.2	swab
EPI_ISL_15739617	patient	swab day 18
EPI_ISL_14518049	BA.2	swab
EPI_ISL_14518038	patient	swab day 26
EPI_ISL_14518039	patient	swab day 32
EPI_ISL_14518137	patient	swab day 47
EPI_ISL_14518040	patient	swab day 55
EPI_ISL_14935895	patient	swab day 60
EPI_ISL_14518101	patient	swab day 76
EPI_ISL_14935908	patient	BAL
EPI_ISL_15251240	patient	swab day 85
EPI_ISL_15251241	patient	swab day 91
EPI_ISL_15251242	patient	swab day 96
EPI_ISL_14935931	patient	BAL day 103
EPI_ISL_15251243	patient	BAL day 111
EPI_ISL_15511842	patient	BAL day 141
EPI_ISL_15511843	patient	swab day 4
EPI_ISL_15719141	patient	BAL day 4
EPI_ISL_15719142	patient	swab day 4
EPI_ISL_11268046	BA.2	swab
EPI_ISL_11268047	BA.2	swab
EPI_ISL_11268048	BA.2	swab
EPI_ISL_11268049	BA.2	swab
EPI_ISL_11268052	BA.2	swab
EPI_ISL_11268053	BA.2	swab
EPI_ISL_11268054	BA.2	swab
EPI_ISL_11268055	BA.2	swab
EPI_ISL_11268058	BA.2	swab
EPI_ISL_11268059	BA.2	swab
EPI_ISL_11268060	BA.2	swab

seq	lineage	specimen
EPI_ISL_11268061	BA.2	swab
EPI_ISL_11268062	BA.2	swab
EPI_ISL_11268063	BA.2	swab
EPI_ISL_11268065	BA.2	swab
EPI_ISL_11268066	BA.2	swab
EPI_ISL_11268067	BA.2	swab
EPI_ISL_11268069	BA.2	swab
EPI_ISL_11268070	BA.2	swab
EPI_ISL_11268071	BA.2	swab
EPI_ISL_11268073	BA.2	swab
EPI_ISL_11268075	BA.2	swab
EPI_ISL_11268076	BA.2	swab
EPI_ISL_11268077	BA.2	swab
EPI_ISL_11268078	BA.2	swab
EPI_ISL_11268079	BA.2	swab
EPI_ISL_11268081	BA.2	swab
EPI_ISL_11268082	BA.2	swab
EPI_ISL_11793120	BA.2	swab
EPI_ISL_11793122	BA.2	swab
EPI_ISL_11793124	BA.2	swab
EPI_ISL_11793125	BA.2	swab
EPI_ISL_11793126	BA.2	swab
EPI_ISL_11793128	BA.2	swab
EPI_ISL_11793194	BA.2	swab
EPI_ISL_11793196	BA.2	swab
EPI_ISL_11793131	BA.2	swab
EPI_ISL_11793133	BA.2	swab
EPI_ISL_11793134	BA.2	swab
EPI_ISL_11793198	BA.2	swab
EPI_ISL_11793199	BA.2	swab
EPI_ISL_11793200	BA.2	swab
EPI_ISL_11793201	BA.2	swab
EPI_ISL_11793202	BA.2	swab
EPI_ISL_11793203	BA.2	swab
EPI_ISL_11793207	BA.2	swab
EPI_ISL_11793208	BA.2	swab
EPI_ISL_11793209	BA.2	swab
EPI_ISL_11793211	BA.2	swab
EPI_ISL_11793212	BA.2	swab
EPI_ISL_11793213	BA.2	swab
EPI_ISL_11793214	BA.2	swab
EPI_ISL_11793216	BA.2	swab
EPI_ISL_11793217	BA.2	swab
EPI_ISL_11793218	BA.2	swab
EPI_ISL_11793220	BA.2	swab
EPI_ISL_11793135	BA.2	swab
EPI_ISL_11793222	BA.2	swab
EPI_ISL_11793223	BA.2	swab
EPI_ISL_11793224	BA.2	swab
EPI_ISL_11793225	BA.2	swab
EPI_ISL_11793145	BA.2	swab
EPI_ISL_11793227	BA.2	swab
EPI_ISL_11793228	BA.2	swab
EPI_ISL_13748025	BA.2	swab
EPI_ISL_13748026	BA.2	swab
EPI_ISL_13748027	BA.2	swab
EPI_ISL_13748028	BA.2	swab
EPI_ISL_11793138	BA.2	swab
EPI_ISL_11793146	BA.2	swab
EPI_ISL_11793147	BA.2	swab
EPI_ISL_11793148	BA.2	swab
EPI_ISL_11793149	BA.2	swab
EPI_ISL_11793150	BA.2	swab
EPI_ISL_11793152	BA.2	swab
EPI_ISL_11793153	BA.2	swab
EPI_ISL_11793139	BA.2	swab

seq	lineage	specimen
EPI_ISL_11793154	BA.2	swab
EPI_ISL_11793141	BA.2	swab
EPI_ISL_11793156	BA.2	swab
EPI_ISL_11793158	BA.2	swab
EPI_ISL_11793159	BA.2	swab
EPI_ISL_11793160	BA.2	swab
EPI_ISL_11793162	BA.2	swab
EPI_ISL_11793163	BA.2	swab
EPI_ISL_11793164	BA.2	swab
EPI_ISL_11793165	BA.2	swab
EPI_ISL_11793166	BA.2	swab
EPI_ISL_11793169	BA.2	swab
EPI_ISL_11793171	BA.2	swab
EPI_ISL_11793173	BA.2	swab
EPI_ISL_11793174	BA.2	swab
EPI_ISL_11793175	BA.2	swab
EPI_ISL_11793176	BA.2	swab
EPI_ISL_11793177	BA.2	swab
EPI_ISL_11793178	BA.2	swab
EPI_ISL_11793179	BA.2	swab
EPI_ISL_11793180	BA.2	swab
EPI_ISL_11793181	BA.2	swab
EPI_ISL_11793182	BA.2	swab
EPI_ISL_11793184	BA.2	swab
EPI_ISL_11793185	BA.2	swab
EPI_ISL_11793187	BA.2	swab
EPI_ISL_11793188	BA.2	swab
EPI_ISL_13748030	BA.2	swab
EPI_ISL_13748031	BA.2	swab
EPI_ISL_13748032	BA.2	swab
EPI_ISL_13748034	BA.2	swab
EPI_ISL_13748036	BA.2	swab
EPI_ISL_13748037	BA.2	swab
EPI_ISL_13748038	BA.2	swab
EPI_ISL_13748039	BA.2	swab
EPI_ISL_13748040	BA.2	swab
EPI_ISL_13748041	BA.2	swab
EPI_ISL_13748042	BA.2	swab
EPI_ISL_13748043	BA.2	swab
EPI_ISL_13748044	BA.2	swab
EPI_ISL_13748045	BA.2	swab
EPI_ISL_13748046	BA.2	swab
EPI_ISL_13748047	BA.2	swab
EPI_ISL_13748049	BA.2	swab
EPI_ISL_13748050	BA.2	swab
EPI_ISL_13748051	BA.2	swab
EPI_ISL_13748052	BA.2	swab
EPI_ISL_13748053	BA.2	swab
EPI_ISL_13748054	BA.2	swab
EPI_ISL_13748055	BA.2	swab
EPI_ISL_13748056	BA.2	swab
EPI_ISL_13748057	BA.2	swab
EPI_ISL_13748068	BA.2	swab
EPI_ISL_13748058	BA.2	swab
EPI_ISL_13748069	BA.2	swab
EPI_ISL_13748059	BA.2	swab
EPI_ISL_13748060	BA.2	swab
EPI_ISL_13748071	BA.2	swab
EPI_ISL_13748072	BA.2	swab
EPI_ISL_13748073	BA.2	swab
EPI_ISL_13748074	BA.2	swab
EPI_ISL_13748075	BA.2	swab
EPI_ISL_13748076	BA.2	swab
EPI_ISL_13748077	BA.2	swab
EPI_ISL_13748078	BA.2	swab
EPI_ISL_13748079	BA.2	swab

Supplementary Table 4 (continued)

seq	lineage	specimen
EPI_ISL_13748080	BA.2	swab
EPI_ISL_13748083	BA.2	swab
EPI_ISL_13748084	BA.2	swab
EPI_ISL_13748090	BA.2	swab
EPI_ISL_13748091	BA.2	swab
EPI_ISL_13748093	BA.2	swab
EPI_ISL_13748094	BA.2	swab
EPI_ISL_13748095	BA.2	swab
EPI_ISL_13748097	BA.2	swab
EPI_ISL_13748110	BA.2	swab
EPI_ISL_13748098	BA.2	swab
EPI_ISL_13748100	BA.2	swab
EPI_ISL_13748111	BA.2	swab
EPI_ISL_13748113	BA.2	swab
EPI_ISL_13748114	BA.2	swab
EPI_ISL_13748137	BA.2	swab
EPI_ISL_13748116	BA.2	swab
EPI_ISL_13748117	BA.2	swab
EPI_ISL_13748118	BA.2	swab
EPI_ISL_13748119	BA.2	swab
EPI_ISL_13748120	BA.2	swab
EPI_ISL_13748121	BA.2	swab
EPI_ISL_13748122	BA.2	swab
EPI_ISL_13748123	BA.2	swab
EPI_ISL_13748124	BA.2	swab
EPI_ISL_13748125	BA.2	swab
EPI_ISL_13748126	BA.2	swab
EPI_ISL_13748130	BA.2	swab
EPI_ISL_13748131	BA.2	swab
EPI_ISL_13748132	BA.2	swab
EPI_ISL_13748133	BA.2	swab
EPI_ISL_13748135	BA.2	swab
EPI_ISL_13748136	BA.2	swab
EPI_ISL_13748138	BA.2	swab
EPI_ISL_13748139	BA.2	swab
EPI_ISL_13748140	BA.2	swab
EPI_ISL_13748141	BA.2	swab
EPI_ISL_13748144	BA.2	swab
EPI_ISL_13748145	BA.2	swab
EPI_ISL_13748147	BA.2	swab
EPI_ISL_13748148	BA.2	swab
EPI_ISL_13748149	BA.2	swab
EPI_ISL_13748150	BA.2	swab
EPI_ISL_13748151	BA.2	swab
EPI_ISL_13748153	BA.2	swab
EPI_ISL_13748154	BA.2	swab
EPI_ISL_13748157	BA.2	swab
EPI_ISL_13748158	BA.2	swab
EPI_ISL_13748159	BA.2	swab
EPI_ISL_13748160	BA.2	swab
EPI_ISL_13748161	BA.2	swab
EPI_ISL_13748162	BA.2	swab
EPI_ISL_13748163	BA.2	swab
EPI_ISL_13748164	BA.2	swab
EPI_ISL_13748165	BA.2	swab
EPI_ISL_15739618	BA.2	swab
EPI_ISL_13748168	BA.2	swab
EPI_ISL_13748170	BA.2	swab
EPI_ISL_13748172	BA.2	swab
EPI_ISL_13748175	BA.2	swab
EPI_ISL_13748178	BA.2	swab
EPI_ISL_15739619	BA.2	swab
EPI_ISL_13748182	BA.2	swab
EPI_ISL_13748184	BA.2	swab
EPI_ISL_13748185	BA.2	swab
EPI_ISL_13748186	BA.2	swab
EPI_ISL_13748187	BA.2	swab
EPI_ISL_13748188	BA.2	swab
EPI_ISL_13748189	BA.2	swab
EPI_ISL_13748191	BA.2	swab
EPI_ISL_13748192	BA.2	swab
EPI_ISL_13748194	BA.2	swab
EPI_ISL_13748195	BA.2	swab
EPI_ISL_13748196	BA.2	swab
EPI_ISL_13748197	BA.2	swab
EPI_ISL_13748198	BA.2	swab
EPI_ISL_13748201	BA.2	swab
EPI_ISL_13748202	BA.2	swab
EPI_ISL_13748204	BA.2	swab
EPI_ISL_13748205	BA.2	swab
EPI_ISL_13748206	BA.2	swab
EPI_ISL_13748207	BA.2	swab
EPI_ISL_13748208	BA.2	swab
EPI_ISL_15739620	BA.2	swab
EPI_ISL_13748210	BA.2	swab
EPI_ISL_15739621	BA.2	swab
EPI_ISL_13748211	BA.2	swab
EPI_ISL_15739622	BA.2	swab
EPI_ISL_15739624	BA.2	swab
EPI_ISL_15739625	BA.2	swab
EPI_ISL_15739626	BA.2	swab
EPI_ISL_15739627	BA.2	swab
EPI_ISL_15739628	BA.2	swab
EPI_ISL_15739629	BA.2	swab
EPI_ISL_14518195	BA.2	swab
EPI_ISL_14518162	BA.2	swab
EPI_ISL_14517992	BA.2	swab
EPI_ISL_14518090	BA.2	swab
EPI_ISL_14518138	BA.2	swab
EPI_ISL_14518109	BA.2	swab
EPI_ISL_14518190	BA.2	swab
EPI_ISL_14518226	BA.2	swab
EPI_ISL_14518085	BA.2	swab
EPI_ISL_14518151	BA.2	swab
EPI_ISL_14518080	BA.2	swab
EPI_ISL_14518087	BA.2	swab
EPI_ISL_14518110	BA.2	swab
EPI_ISL_14935962	BA.2	swab
EPI_ISL_15409727	BA.2	swab
EPI_ISL_15409751	BA.2	swab

Supplementary Table 5 In this study analyzed CD8+ and CD4+ T cell epitopes

epitope	amino acid sequence	pre-described vs predicted
B*27/N_{9-17}	QRNAPRITF	pre-described
B*40/$N_{322-331}$	MEVTPSGTWL	pre-described
A*03/$N_{361-369}$	KTFPPTEPK	pre-described
A*03/$N_{361-370}$	KTFPPTEPKK	pre-described
B*40/$M_{136-145}$	SELVIGAVIL	pre-described
A*01/$M_{171-179}$	ATSRTLSYY	pre-described
A*01/$ORF3a_{207-215}$	FTSDYYQLY	pre-described
A*03/$S_{378-386}$	KCYGVSPTK	pre-described
A*01/$S_{865-873}$	LTDEMIAQY	pre-described
A*03/$ORF1ab_{808-816}$	VTNNTFTLK	pre-described
A*01/$ORF1ab_{1321-1329}$	PTDNYITTY	pre-described
A*01/$ORF1ab_{1636-1646}$	HTTDPSFLGRY	pre-described
A*01/$ORF1ab_{1637-1646}$	TTDPSFLGRY	pre-described
A*01/$ORF1ab_{1889-1899}$	CTEIDPKLDNY	pre-described
A*01/$ORF1ab_{4082-4091}$	NTCDGTTFTY	pre-described
A*01/$ORF1ab_{4163-4171}$	CTDDNALAY	pre-described
A*01/$ORF1ab_{4163-4172}$	CTDDNALAYY	pre-described
A*01/$ORF1ab_{5130-5138}$	DTDFVNEFY	pre-described
A*03/$ORF1ab_{5533-5542}$	VVYRGTTTYK	pre-described
B*40/$ORF1ab_{6219-6228}$	IEYPIIGDEL	pre-described
DRB1*04/15/$ORF1ab_{1801-1815}$	ESPFVMMSAPPAQYE	predicted, non-promiscuous
$ORF1ab_{4227-4241}$	YLYFIKGLNNLNRGM	predicted, promiscuous
$ORF1ab_{5019-5033}$	PNMLRIMASLVLARK	predicted, promiscuous
$ORF1ab_{5974-5988}$	MTYRRLISMMGFKMN	predicted, promiscuous
$ORF1ab_{6987-7001}$	WWTAFVTNVNASSSE	predicted, promiscuous
DRB1*03/15/$S_{236-250}$	TRFQTLLALHRSYLT	predicted, non-promiscuous
DRB1*04/15/M_{99-113}	SFRLFARTRSMWSFN	predicted, non-promiscuous
