# Supplementary material for: SARS-CoV-2 infection dynamics in a MHCI-mismatched lung transplant recipient
Source: Nat Commun. 2025 Sep 16;16:8292. doi: 10.1038/s41467-025-63681-y (PMC12441153; doi:10.1038/s41467-025-63681-y)
Supplement: Supplementary file 2 — Reporting Summary [file 41467_2025_63681_MOESM2_ESM.pdf]

nature portfolio

Corresponding author(s): Björn C. Frye

Last updated by author(s): Jul 18, 2025

Reporting Summary

Nature Portfolio wishes to improve the reproducibility of the work that we publish. This form provides structure for consistency and transparency in reporting. For further information on Nature Portfolio policies, see our Editorial Policies and the Editorial Policy Checklist.

Statistics

For all statistical analyses, confirm that the following items are present in the figure legend, table legend, main text, or Methods section.

n/a	Confirmed	
[ ]	[x]	The exact sample size (n) for each experimental group/condition, given as a discrete number and unit of measurement
[ ]	[x]	A statement on whether measurements were taken from distinct samples or whether the same sample was measured repeatedly
[ ]	[x]	The statistical test(s) used AND whether they are one- or two-sided<br>*Only common tests should be described solely by name; describe more complex techniques in the Methods section.*
[x]	[ ]	A description of all covariates tested
[x]	[ ]	A description of any assumptions or corrections, such as tests of normality and adjustment for multiple comparisons
[ ]	[x]	A full description of the statistical parameters including central tendency (e.g. means) or other basic estimates (e.g. regression coefficient) AND variation (e.g. standard deviation) or associated estimates of uncertainty (e.g. confidence intervals)
[ ]	[x]	For null hypothesis testing, the test statistic (e.g. F, t, r) with confidence intervals, effect sizes, degrees of freedom and P value noted<br>*Give P values as exact values whenever suitable.*
[x]	[ ]	For Bayesian analysis, information on the choice of priors and Markov chain Monte Carlo settings
[x]	[ ]	For hierarchical and complex designs, identification of the appropriate level for tests and full reporting of outcomes
[x]	[ ]	Estimates of effect sizes (e.g. Cohen's d, Pearson's r), indicating how they were calculated

Our web collection on statistics for biologists contains articles on many of the points above.

Software and code

Policy information about availability of computer code

Data collection	-Mutational prevalence:<br>To analyze the global number of sequences that harbor the single or combinatorial spike mutations (S:K356T, S:L368I, S:T385I), GISAID was accessed with the outbreakinfo R package on the 13.02.2023 including all sequences between the 12.01.2021 and 24.10.2022 (~6.9 million sequences.<br><br>-BA.2 consensus mutations:<br>To calculate BA.2 consensus mutations, we downloaded 4992 BA.2 sequences from Baden-Wuerttemberg, Germany between April and December 2022 (GISAID Identifier: EPI_SET_230216es, doi: 10.55876/gis8.230216es).<br><br>-T cell analyses:<br>Multiparametric flow cytometry data were collected by FACSDiva software and CytExpert Software.<br><br>-clinical data:<br>clinical data were extracted from medical charts and stored in password-protected data base for further analysis.
Data analysis	- Statistical analysis:<br>GraphPad Prism (v.8.4.2), R (v.4.2.1) using Rstudio ("Prairie Trillium") or python3.9<br><br>- Variant frequency visualization:<br>github.com/jonas-fuchs/SARS-CoV-2-analyses (v.1.1, https://doi.org/10.5281/zenodo.7692398).

- Protein structure visualization:
UCSF ChimeraX version: 1.1 (2020-09-09).

- Analysis of NGS data:
SARS-CoV-2 NGS data was analyzed on the cloud computing bioinformatic platform Galaxy (usegalaxy.eu, covid19.galaxyproject.org/artic/) using the following pipline: fastqs were preprocessed with fastp (v.0.20.1) and mapped using BWA-MEM (v.0.7.17), ARTIC primer sequences were trimmed using ivar trim (v1.9), SNPs and INDELs were called with lofreq (v2.1.5) and annotated with snpeff (v.4.3.1). Consensus sequences were generated with bcftools (v.1.10).

- Phylogenetic tree:
MAFFT (v.7.45), IQ-TREE multicore version (v2.1.2)
Pylogenetic tree visualization: R packages ggtree v2.2.4, treeio (v1.12.0) and ggplot2 (v3.3.3)

- Mutational profiling:
(2020-09-09).Mutational profiles were determined with covsonar v.1.1.8 (https://github.com/rki-mf1/covsonar) and a lightweight python script (https://github.com/jonas-fuchs/covsonar_con_mut).

-Sequence homology analyses:
Sequence homology analyses were performed in Geneious Prime 2022.0.2 (https://www.geneious.com/) using Clustal Omega 1.2.2 alignment with default settings.

-Analyses of flow cytometry data:
Multiparametric flow cytometry data were analyzed using FlowJo software version 10.7.1 (Treestar, Becton Dickinson).

For manuscripts utilizing custom algorithms or software that are central to the research but not yet described in published literature, software must be made available to editors and reviewers. We strongly encourage code deposition in a community repository (e.g. GitHub). See the Nature Portfolio guidelines for submitting code & software for further information.

Data

Policy information about availability of data

All manuscripts must include a data availability statement. This statement should provide the following information, where applicable:

- Accession codes, unique identifiers, or web links for publicly available datasets
- A description of any restrictions on data availability
- For clinical datasets or third party data, please ensure that the statement adheres to our policy

- All necessary data and information are given in the paper.

- Consensus sequences have been deposited in the GISAID database (www.gisaid.org) (Supplementary Table 2). Raw data has been deposited in the European Nucleotide Archive (ENA) under the study accession number: PRJEB71389.

- The sequence used in this study as the SARS-CoV-2 reference genome has the GenBank [https://www.ncbi.nlm.nih.gov/genbank/] accession: NC_045512 (Wuhan-Hu-1).

- The pdb database accession numbers [https://www.rcsb.org/] that were used for structural analysis and visualization are: 7XIX, 7XB0, 7X1M, 7L7E

The raw values for charts and graphs are available in the Source Data file whenever possible. All requests for additional raw (especially flow cytometry data) and materials are promptly reviewed by the University of Freiburg Center for Technology Transfer to verify if the request is subject to any intellectual property or confidentiality obligations. Donor-related data not included in the paper were generated as part of clinical examination and may be subject to donor confidentiality. Any data and materials that can be shared will be released via a Material Transfer Agreement. Source data are provided with this paper.

Research involving human participants, their data, or biological material

Policy information about studies with human participants or human data. See also policy information about sex, gender (identity/presentation), and sexual orientation and race, ethnicity and racism.

Reporting on sex and gender	A single patient with caucasian ancestry and male sex is reported.
Reporting on race, ethnicity, or other socially relevant groupings	Healthy donor samples were included as controls (n=2 males (37 and 43 years); n=3 females (24, 25 and 27 years)
Population characteristics	*Describe the covariate-relevant population characteristics of the human research participants (e.g. age, genotypic information, past and current diagnosis and treatment categories). If you filled out the behavioural & social sciences study design questions and have nothing to add here, write "See above."*
Recruitment	n.a.
Ethics oversight	Written informed consent was obtained from patient and healthy donors. The study was conducted in accordance to federal

guidelines, local ethics committee regulations (Albert-Ludwigs-Universität, Freiburg, Germany; vote: 322/20, 10/03, 21-1135 and 383/19) and the Declaration of Helsinki (1975).

Note that full information on the approval of the study protocol must also be provided in the manuscript.

Field-specific reporting

Please select the one below that is the best fit for your research. If you are not sure, read the appropriate sections before making your selection.

[x] Life sciences [ ] Behavioural & social sciences [ ] Ecological, evolutionary & environmental sciences

For a reference copy of the document with all sections, see nature.com/documents/nr-reporting-summary-flat.pdf

Life sciences study design

All studies must disclose on these points even when the disclosure is negative.

Sample size	No sample size calculation was performed. Sample sizes were chosen based in the minimum number of independent experiments that allow statistical evaluation (n=3).
Data exclusions	No data were excluded.
Replication	Data were reproduced at least as biological triplicates. Diagnostic assays (ELISAs, qPCR of respiratory samples) were done once using accredited assays due to the limited availability of the individual samples.
Randomization	No randomization was applied as no group allocation was carried out.
Blinding	No blinding was caried out. Blinding was not needed because no group allocation was carried out and no subjective paramenters were measured.

Reporting for specific materials, systems and methods

We require information from authors about some types of materials, experimental systems and methods used in many studies. Here, indicate whether each material, system or method listed is relevant to your study. If you are not sure if a list item applies to your research, read the appropriate section before selecting a response.

Materials & experimental systems

n/a	Involved in the study	
[ ]	[x]	Antibodies
[ ]	[x]	Eukaryotic cell lines
[ ]	[ ]	Palaeontology and archaeology
[ ]	[ ]	Animals and other organisms
[ ]	[ ]	Clinical data
[ ]	[ ]	Dual use research of concern
[ ]	[ ]	Plants

Methods

n/a	Involved in the study	
[ ]	[ ]	ChIP-seq
[ ]	[x]	Flow cytometry
[ ]	[ ]	MRI-based neuroimaging

Antibodies

Antibodies used

BD Biosciences:
anti-CD95-PE (DX2, 1:16.7), mouse, LOT 3114254, Cat# 340480
anti-CD95-BV421 (DX2, 1:16.7), mouse, LOT 286661, Cat# 562616
anti-CD103-FITC (Ber-ACT8, 1:20), mouse, LOT 0079918-1, Cat# 550259
anti-IFN-γ-FITC (25723.11, 1:8), mouse, LOT 4260955, Cat# 340449
anti-CD8-APC (SK1, 1:200), mouse, LOT 4213691, Cat# 345775
anti-CD8-BV510 (SK1, 1:100), mouse, LOT 4134058, Cat# 563919
anti-CD8-BV421 (RPA-T8, 1:200), mouse, LOT 1292699, Cat# 562428
anti-CD45-PE-Cy5 (HI30, 1:50), mouse IgG1, Cat# 555484
anti-CD3-FITC (UCHT1, 1:50), mouse IgG1, Cat# 555332
anti-CD25-PE (M-A251, 1:50), mouse IgG1, Cat# 555432
anti-CD107a-APC (H4A3, 1:100), mouse, LOT 5149854, Cat# 560664
anti-TNF-PE-Cy7 (MAb11, 1:50), mouse, LOT 3292259, Cat# 557647
anti-CD4-BUV395 (SK3, 1:100), mouse, LOT 2332927, Cat# 563550

BioLegend:
anti-CCR7-PE-Dazzle594 (G043H7, 1:50), mouse, LOT B348534, Cat# 353236

anti-CD8-BV650 (RPA-T8, 1:200), mouse, LOT B361475, Cat# 301042
anti-CD20-APC Fire 750 (2H7, 1:50), mouse IgG2b, Cat# 302358
anti-CD56-Pacific Blue (MEM188, 1:50), mouse IgG2a, Cat# 304629
anti-CD57-Pacific Blue (HNK1, 1:50), mouse IgGM, Cat# 359608

Thermo Fisher Scientific:
anti-CD14-APC-eFluor780 (61D3, 1:800), mouse, LOT 2730940, Cat# 47-0149-42
anti-CD19-APC-eFluor780 (HIB19, 1:800), mouse, LOT 2892943, Cat# 47-0199
anti-CD69-PE-Cy7 (FN50, 1:50), mouse, LOT 2077748, Cat# 25-0699-42
anti-CD4-eFlour450 (RPA-T4, 1:250), mouse, LOT 2526326, Cat# 48-0049-42
anti-HLA-A3-APC (GAP.A3, 1:25), mouse, LOT 28968, Cat# 17-5754
Viability Dye (APC-eFluor780, 1:800) LOT 3154429, Cat# 65-0865-14
Viability Dye (eFluor506, 1:300) LOT 2220742, Cat# 65-0866-14

Immunotools:
anti-CD4-APC (MEM-241, 1:50), mouse IgG2b, Cat# 21270046X2
anti-CD8-PE (UCHT-4, 1:50), mouse IgG2a, Cat# 21620084X2
anti-HLA-DR-APC (LT-DR, 1:50), mouse IgG2a, Cat# 21388996

Validation

All antibodies were obtained from commercial vendors and we based specificity on descriptions and information provided in corresponding data sheets available and provided by the manufacturers. Standardized analysis in different cohorts, antibody titration on PBMCs including unstained controls, comparisons of different antibody clones and conjugates, and validated by publications:
CCR7, clone G043H7, flow cytometry: antibody titration on PBMCs; control clone 3D12; validated with respect to differential expression of naïve and non-naïve T cell subpopulations (PMID: 33296701)
CD8, clone SK1 and RPA-T8, flow cytometry: antibody titration on PBMCs; control clone GHI/75; using B cells as negative control (PMID: 35654046, 35572194, 35474871)
CD4, clone SK3 and RPA-T4, flow cytometry: antibody titration on PBMCs; control clone L200; using B cells as negative control (PMID: 1713585)
CD14, clone 61D3, flow cytometry: antibody titration on PBMCs; control clones M5E2 and MϕP9; using T cell populations as negative control (PMID: 34321489)
CD19, clone HIB19, flow cytometry: antibody titration on PBMCs; control clone SJ25C1; using T cell populations as negative control (PMID: 34133950)
CD95, clone DX2, flow cytometry: Titration on fresh PBMCs and in vitro activated B cells; validated with respect to differential expression of naïve and non-naïve T cell subpopulations (PMID: 7523573)
CD107a, clone H4A3: antibody titration on PBMCs; validated with respect to differential expression of activated and non-activated T cell subpopulations (PMID: 34561618)
IFNɣ, clone 25723.11, flow cytometry: antibody titration on PBMCs; control clone 4S.B3; validated with respect to differential expression of activated and non-activated T cell subpopulations (PMID: 36456734)
CD103, clone Ber-ACT8, flow cytometry: antibody titration on tissue-derived cells; validated with respect to differential expression of tissue- and blood-derived T cell populations (PMID: 34224909)
CD69, clone FN50, flow cytometry: antibody titration on tissue-derived cells; validated with respect to differential expression of tissue- and blood-derived T cell populations (PMID: 35985993)
TNF, clone MAb11, flow cytometry: antibody titration on PBMCs; validated with respect to differential expression of activated and non-activated T cell subpopulations (PMID: 37468623)
Viability Dyes were titrated on PBMCs; validated with respect to differential staining of live and dead cell populations and by the manufacturer (https://www.thermofisher.com/order/catalog/product/de/en/65-0865-14)

All antibodies for BAL analysis were obtained from commercial vendors and we based specificity on descriptions and information provided in corresponding data sheets available and provided by the manufacturers. The following antibodies are routinely used in our and other laboratories to characterize lymphocyte subsets in bronchoalveolar lavage (PMID: 20442436; PMID: 26649486; PMID: 29449421):
CD45 identifies bone marrow-derivet immune cells. In combination with side-scatter used to differentiate lymphocytes from neutrophile (lower CD45 expression in BAL) and alveolar macrophages (higher side scatter)
CD3 used to identify T cells (ISBN-10: 0192618679)
CD4 used to identify T helper cells (ISBN-10: 0192618679)
CD8 used to identify cytotoxic T cells (ISBN-10: 0192618679)
CD20 is expressed throughout B cell development, up until their differentiation into plasma cells. Thus, it can be used as a pan-B cell identifier (ISBN-10: 0192618679)
CD25 is the high-affinity IL-2 receptor and indicates freshly activated T cells (PMID: 10197730)
CD56 is a protein expressed on NK and NKT cells. In combination with CD3 it is used to differentiate NK cells (CD56+/CD3-) from NKT cells (CD56+/CD3+) (PMID: 15994392)
CD57 is expressed on subsets of NK and CD8+ cells. However, in BAL it might be expressed on CD4+ and CD8+ T cells. CD57high expression indicates terminally differentiated or senescent cells (PMID: 17416406)
HLA-DR HLA-DR is expressed on repeatedly stimulated cells indicating long lasting inflammatory processes. (PMID: 8519382)

Eukaryotic cell lines

Policy information about cell lines and Sex and Gender in Research

Cell line source(s)	VeroE6 cells (ATCC CRL-1586) and), Calu-3 cells (ATCC-HTB-5)
Authentication	None of the cell lines were authenticated
Mycoplasma contamination	all cell lines were tested monthly negative for mycoplasma
Commonly misidentified lines (See ICLAC register)	no commonly misidentified cell lines were used in the study

Palaeontology and Archaeology

Specimen provenance	*Provide provenance information for specimens and describe permits that were obtained for the work (including the name of the issuing authority, the date of issue, and any identifying information). Permits should encompass collection and, where applicable, export.*
Specimen deposition	*Indicate where the specimens have been deposited to permit free access by other researchers.*
Dating methods	*If new dates are provided, describe how they were obtained (e.g. collection, storage, sample pretreatment and measurement), where they were obtained (i.e. lab name), the calibration program and the protocol for quality assurance OR state that no new dates are provided.*

☐ Tick this box to confirm that the raw and calibrated dates are available in the paper or in Supplementary Information.

Ethics oversight	*Identify the organization(s) that approved or provided guidance on the study protocol, OR state that no ethical approval or guidance was required and explain why not.*

Note that full information on the approval of the study protocol must also be provided in the manuscript.

Animals and other research organisms

Policy information about studies involving animals; ARRIVE guidelines recommended for reporting animal research, and Sex and Gender in Research

Laboratory animals	*For laboratory animals, report species, strain and age OR state that the study did not involve laboratory animals.*
Wild animals	*Provide details on animals observed in or captured in the field; report species and age where possible. Describe how animals were caught and transported and what happened to captive animals after the study (if killed, explain why and describe method; if released, say where and when) OR state that the study did not involve wild animals.*
Reporting on sex	*Indicate if findings apply to only one sex; describe whether sex was considered in study design, methods used for assigning sex. Provide data disaggregated for sex where this information has been collected in the source data as appropriate; provide overall numbers in this Reporting Summary. Please state if this information has not been collected. Report sex-based analyses where performed, justify reasons for lack of sex-based analysis.*
Field-collected samples	*For laboratory work with field-collected samples, describe all relevant parameters such as housing, maintenance, temperature, photoperiod and end-of-experiment protocol OR state that the study did not involve samples collected from the field.*
Ethics oversight	*Identify the organization(s) that approved or provided guidance on the study protocol, OR state that no ethical approval or guidance was required and explain why not.*

Note that full information on the approval of the study protocol must also be provided in the manuscript.

Clinical data

Policy information about clinical studies

All manuscripts should comply with the ICMJEguidelines for publication of clinical research and a completedCONSORT checklist must be included with all submissions.

Clinical trial registration	*Provide the trial registration number from ClinicalTrials.gov or an equivalent agency.*
Study protocol	*Note where the full trial protocol can be accessed OR if not available, explain why.*
Data collection	Data collection of patient-related data was performed retrospectively. Relevant clinical data were extracted from medical charts and stored anonymized in a password-protected database.
Outcomes	*Describe how you pre-defined primary and secondary outcome measures and how you assessed these measures.*

Dual use research of concern

Policy information about dual use research of concern

Hazards

Could the accidental, deliberate or reckless misuse of agents or technologies generated in the work, or the application of information presented in the manuscript, pose a threat to:

No	Yes	
☒	☐	Public health
☒	☐	National security
☒	☐	Crops and/or livestock
☒	☐	Ecosystems
☒	☐	Any other significant area

Experiments of concern

Does the work involve any of these experiments of concern:

No	Yes	
☒	☐	Demonstrate how to render a vaccine ineffective
☒	☐	Confer resistance to therapeutically useful antibiotics or antiviral agents
☒	☐	Enhance the virulence of a pathogen or render a nonpathogen virulent
☒	☐	Increase transmissibility of a pathogen
☒	☐	Alter the host range of a pathogen
☒	☐	Enable evasion of diagnostic/detection modalities
☒	☐	Enable the weaponization of a biological agent or toxin
☒	☐	Any other potentially harmful combination of experiments and agents

Plants

Seed stocks	*Report on the source of all seed stocks or other plant material used. If applicable, state the seed stock centre and catalogue number. If plant specimens were collected from the field, describe the collection location, date and sampling procedures.*
Novel plant genotypes	*Describe the methods by which all novel plant genotypes were produced. This includes those generated by transgenic approaches, gene editing, chemical/radiation-based mutagenesis and hybridization. For transgenic lines, describe the transformation method, the number of independent lines analyzed and the generation upon which experiments were performed. For gene-edited lines, describe the editor used, the endogenous sequence targeted for editing, the targeting guide RNA sequence (if applicable) and how the editor was applied.*
Authentication	*Describe any authentication procedures for each seed stock used or novel genotype generated. Describe any experiments used to assess the effect of a mutation and, where applicable, how potential secondary effects (e.g. second site T-DNA insertions, mosiacism, off-target gene editing) were examined.*

ChIP-seq

Data deposition

☐ Confirm that both raw and final processed data have been deposited in a public database such as GEO.

☐ Confirm that you have deposited or provided access to graph files (e.g. BED files) for the called peaks.

Data access links<br>*May remain private before publication.*	*For "Initial submission" or "Revised version" documents, provide reviewer access links. For your "Final submission" document, provide a link to the deposited data.*
Files in database submission	*Provide a list of all files available in the database submission.*
Genome browser session<br>(e.g. UCSC)	*Provide a link to an anonymized genome browser session for "Initial submission" and "Revised version" documents only, to enable peer review. Write "no longer applicable" for "Final submission" documents.*

Methodology

Replicates	*Describe the experimental replicates, specifying number, type and replicate agreement.*
Sequencing depth	*Describe the sequencing depth for each experiment, providing the total number of reads, uniquely mapped reads, length of reads and whether they were paired- or single-end.*
Antibodies	*Describe the antibodies used for the ChIP-seq experiments; as applicable, provide supplier name, catalog number, clone name, and lot number.*
Peak calling parameters	*Specify the command line program and parameters used for read mapping and peak calling, including the ChIP, control and index files used.*
Data quality	*Describe the methods used to ensure data quality in full detail, including how many peaks are at FDR 5% and above 5-fold enrichment.*
Software	*Describe the software used to collect and analyze the ChIP-seq data. For custom code that has been deposited into a community repository, provide accession details.*

Flow Cytometry

Plots

Confirm that:

- [x] The axis labels state the marker and fluorochrome used (e.g. CD4-FITC).
- [x] The axis scales are clearly visible. Include numbers along axes only for bottom left plot of group (a 'group' is an analysis of identical markers).
- [x] All plots are contour plots with outliers or pseudocolor plots.
- [x] A numerical value for number of cells or percentage (with statistics) is provided.

Methodology

Sample preparation	Cells were isolated from blood and bronchoalveolar lavage fluid and subsequently prepared for flow cytometry or in vitro expansion as described in the methods section.
Instrument	FACSCanto II (BD, Germany), LSRFortessa (BD, Germany) and CytoFLEX (Beckman Coulter, Germany)
Software	FlowJo v10.7.1 (Treestar)
Cell population abundance	Abundance of SARS-CoV-2-specific T cells are low (<10^-4 %)
Gating strategy	CD8+ T cells: Lymphocytes gated on FSC-A and SSC-A, doublet exclusion on FSC-A and FSC-H and SSC-A and SSC-H, exclusion of dead cells, B cells and monocytes, gating on CD8+ cells, gating of SARS-CoV-2-specific CD8+ T cells via tetramers described in methods part.<br>CD4+ T cells: Lymphocytes gated on FSC-A and SSC-A, doublet exclusion on FSC-A and FSC-H and SSC-A and SSC-H, exclusion of dead cells, B cells and monocytes, gating on CD4+ cells, gating of SARS-CoV-2-specific CD4+ T cells via tetramers described in methods part.

- [x] Tick this box to confirm that a figure exemplifying the gating strategy is provided in the Supplementary Information.

Magnetic resonance imaging

Experimental design

Design type	*Indicate task or resting state; event-related or block design.*
Design specifications	*Specify the number of blocks, trials or experimental units per session and/or subject, and specify the length of each trial or block (if trials are blocked) and interval between trials.*
Behavioral performance measures	*State number and/or type of variables recorded (e.g. correct button press, response time) and what statistics were used to establish that the subjects were performing the task as expected (e.g. mean, range, and/or standard deviation across subjects).*

Acquisition

Imaging type(s) — *Specify: functional, structural, diffusion, perfusion.*

Field strength — *Specify in Tesla*

Sequence & imaging parameters — *Specify the pulse sequence type (gradient echo, spin echo, etc.), imaging type (EPI, spiral, etc.), field of view, matrix size, slice thickness, orientation and TE/TR/flip angle.*

Area of acquisition — *State whether a whole brain scan was used OR define the area of acquisition, describing how the region was determined.*

Diffusion MRI ☐ Used ☐ Not used

Preprocessing

Preprocessing software — *Provide detail on software version and revision number and on specific parameters (model/functions, brain extraction, segmentation, smoothing kernel size, etc.).*

Normalization — *If data were normalized/standardized, describe the approach(es): specify linear or non-linear and define image types used for transformation OR indicate that data were not normalized and explain rationale for lack of normalization.*

Normalization template — *Describe the template used for normalization/transformation, specifying subject space or group standardized space (e.g. original Talairach, MNI305, ICBM152) OR indicate that the data were not normalized.*

Noise and artifact removal — *Describe your procedure(s) for artifact and structured noise removal, specifying motion parameters, tissue signals and physiological signals (heart rate, respiration).*

Volume censoring — *Define your software and/or method and criteria for volume censoring, and state the extent of such censoring.*

Statistical modeling & inference

Model type and settings — *Specify type (mass univariate, multivariate, RSA, predictive, etc.) and describe essential details of the model at the first and second levels (e.g. fixed, random or mixed effects; drift or auto-correlation).*

Effect(s) tested — *Define precise effect in terms of the task or stimulus conditions instead of psychological concepts and indicate whether ANOVA or factorial designs were used.*

Specify type of analysis: ☐ Whole brain ☐ ROI-based ☐ Both

Statistic type for inference — *Specify voxel-wise or cluster-wise and report all relevant parameters for cluster-wise methods.*

(See Eklund et al. 2016)

Correction — *Describe the type of correction and how it is obtained for multiple comparisons (e.g. FWE, FDR, permutation or Monte Carlo).*

Models & analysis

n/a	Involved in the study
☒	☐ Functional and/or effective connectivity
☒	☐ Graph analysis
☒	☐ Multivariate modeling or predictive analysis
